# Bipolar disorders and Wilson’s disease

**DOI:** 10.1186/1471-244X-12-52

**Published:** 2012-05-30

**Authors:** Mauro Giovanni Carta, Orazio Sorbello, Maria Francesca Moro, Krishna M Bhat, Enrico Demelia, Alessandra Serra, Gioia Mura, Federica Sancassiani, Mario Piga, Luigi Demelia

**Affiliations:** 1 University of Cagliari, Italy, Cagliari, Italy; 2 Department of Neuroscience and Cell Biology, University of Texas Medical Branch, Galveston, TX, USA

## Abstract

**Background:**

The aim of this study was to determine the risk for Bipolar Disorder (BD) in Wilson’s disease (WD) and to measure the impaired Quality of Life (QL) in BD with WD using standardized psychiatric diagnostic tools and a case control design.

**Methods:**

This was a case control study. The cases were 23 consecutive patients with WD treated at the University Hospital in Cagliari, Italy, and the controls were 92 sex- and age-matched subjects with no diagnosis of WD who were randomly selected from a database used previously for an epidemiological study. Psychiatric diagnoses according to DSM-IV criteria were determined by physicians using structured interview tools (ANTAS-SCID). QL was measured by means of SF-12.

**Results:**

Compared to controls, WD patients had lower scores on the SF-12 and higher lifetime prevalence of DSM-IV major depressive disorders (OR = 5.7, 95% CI 2.4–17.3) and bipolar disorders (OR = 12.9, 95% CI 3.6–46.3). BD was associated with lower SF-12 in WD patients.

**Conclusions:**

This study was the first to show an association between BD and WD using standardized diagnostic tools and a case control design. Reports in the literature about increased schizophrenia-like psychosis in WD and a lack of association with bipolar disorders may thus have been based on a more inclusive diagnosis of schizophrenia in the past. Our findings may explain the frequent reports of loss of emotional control, hyperactivity, loss of sexual inhibition, and irritability in WD patients. This study was limited by a small sample size.

## Background

Wilson’s disease (WD) is an inherited autosomal recessive disorder that affects copper metabolism. It is caused by mutations in a gene on chromosome 13 that encodes ATP 7B, an adenosine triphosphatase involved in copper transportation across cell membranes [1,2]. The prevalence of WD is approximately 1:30,000, with a carrier prevalence of 1:90 [3]. Prompt diagnosis and treatment is critical; the disease is fatal unless treated, and effective treatment is available [4]. The clinical manifestations of WD result from gradual accumulation of free copper in tissues, which can damage many organs. Most WD patients have signs of liver and central nervous system involvement. WD at presentation can have diverse clinical features, including hepatic, neurological, and psychiatric manifestations. WD is usually diagnosed in patients between 6 and 8 years of age when symptoms first become clear [5]. Onset in patients >40 years is rare [6].

The psychiatric manifestations of WD have been reported frequently since the original paper by S.A.K Wilson was published in 1912 [7]. In his report, Wilson describes psychiatric symptoms in 8 of 12 cases, and “schizophrenia-like psychosis” in 2 of the cases. Other reports describe a wide spectrum of psychiatric disorders, including cognitive impairment [8], dementia [9], mental retardation [10], anxiety [11], “schizophrenia-like states” [12], and behavior abnormalities and personality disorders [13].

Depression is extremely common in WD patients, affecting 30–60% of those with WD [13,14]. An association between the degree of disability and the presence of depression has not been established, however. Furthermore, the prevalence of depression is higher in WD than in other chronic disabling diseases, such as rheumatic arthritis, even when there is a similar level of disability [15].

Other mood disorders, such as hypomania and frank mania have also been reported in WD in recent years in two large case series [16,17] and there is some evidence of bipolar disorder in WD in descriptions of two cases in which a manic episode preceded the onset of neurological symptoms [18,19].

Costa-Machado and colleagues [18] suggested that there may be a higher association between bipolar disorder and WD than has been reported in literature.

A study confirming the case series evidence with using standardized diagnostic criteria and a matched case controls methodology will be of interest in terms of clinical practice, and probably in accordance with the broadening of the concept of bipolar disorder in present day psychiatry [20]. Indeed, recent studies indicate that higher levels of copper, similar to several other trace elements such as zinc, cadmium, and thallium, may play a role in the pathophysiology of bipolar disorder, which is consistent with the neurodegenerative hypothesis of such a disorders [21].

The objectives of this study were to determine the frequency of bipolar disorders and mood disorders in a consecutive series of WD inpatients using standardized diagnostic tools and to compare the risk for bipolar disorder in WD patients with the risk in controls. Data were collected from the controls for use in a large epidemiological study [22,23].

The subjective perception of quality of life is a construct that is very relevant to measures of outcomes in chronic disease [24,25], particularly in patients with diseases that impact heavily the daily life of the affected patients and their families [26], such as WD. Thus, the secondary objective of this study was to evaluate the impairment of quality of life and the relationship between mood disorders and impaired quality of life in WD.

## Methods

### Study design

This was a case control study.

### Groups

The cases were 23 consecutive WD patients seen between January and September 2010 at the Gastroenterology Unit at the University Hospital in Cagliari, Italy, a center of excellence that cares WD patients from all regions in Italy. The controls included 92 subjects with no diagnosis of WD who were randomly selected from a database used for an epidemiological study of health conditions in Italy [22,23]. The selection of sex- age-and residence matched controls from the 3498-subject database from six Italian regions (2 from the North, 1 from the Center, 2 from the South plus Sardinia) was performed using a randomized block design. A block was constructed for each case that included all eligible age-matched (± 1 year),sex-matched and residence-matched (North 1 Case 4.3%, South 1 Case 4.3%, Center 0% Italy and Sardinia 21 Cases = 82.6%) controls in the database. Four individuals per block were extracted for each case, automatically excluding them from remaining blocks.

### Psychiatric diagnosis interview, tools, and psychiatric assessment

The psychiatric interviews were conducted using several standardized tools. First, we used a standard form to record basic demographic data. Second, the “Advanced Neuropsychiatric Tools and Assessment Schedule” (ANTAS) [22], a semi-structured clinical interview derived in part from the non-patient version (SCIDI/NP) for DSM-IV [27] (First et al. 2001), was used to assess the presence of full or sub-threshold psychiatric disorders. The ANTAS tool was administered by physicians, which was necessary for it to be administered according to the study protocol. A preliminary reliability study of the diagnoses derived from ANTAS and SCID was carried out, and the results were published previously. The reliability in terms of mood and anxiety diagnoses using ANTAS vs. SCID was measured, and the mean K was 0.85 [20]. Third, the Mood Disorder Questionnaire (MDQ; Italian version) [28] was used to assess bipolar spectrum disorders. The adopted MDQ cut off was 7 (Bipolar Cases identified by score of 7 or more) [28].

Fourth, quality of life was evaluated with the Short Form Health Survey (SF-12) [29]. The SF-12 includes the following dimensions: physical activity, physical health limitations on roles or activities, emotional state, physical pain, self-evaluation of general state of health, vitality, social activity, and mental health. The period of measurement was the month prior to evaluation. Higher scores on the SF-12 correspond to better conditions and quality of life. Finally, the interviewers asked the interviewees to show them the packages of drugs they used and then retained all of the psychotropic drug box covers in a folder provided for this purpose.

### Diagnosis of WD

The diagnosis for each WD patient was made by evaluating the clinical symptoms, laboratory test results, and results of mutation analysis [30]. Confirmation of the clinical diagnosis included decreased serum ceruloplasmin concentration, high serum non-ceruloplasmin-bound copper concentration, low serum copper concentration, elevated 24-h urinary copper excretion, elevated hepatic copper content (>250 μg/g dry weight), the presence of Kayser-Fleischer rings by slit lamp examination, and ATP7B mutation.

Liver function tests were performed, including alanine aminotransferase (ALT), aspartate aminotransferase (AST), and g-glutamyl transpeptidase (GGT) tests. Quantitative determination of serum ceruloplasmin concentrations was performed using automated clinical chemistry analyzers (Array Protein System 360, Beckman Instruments, California, USA) using a nephelometric assay. The reference range for serum ceruloplasmin is between 200–600 mg/liter. Serum copper and urinary copper concentrations were measured using an inductively coupled plasma optical emission spectrometer. The reference range for serum copper is 10–22 μmol/liter. In WD, the urinary copper output is >1.5 μmoles/24 h. Liver copper concentration in dried liver tissue was measured by flame atomic absorption spectroscopy (Instrumentation Laboratory Video 22). Liver copper content >250 μg/g dry weight is diagnostic for WD.

### Screening controls for WD

During the interviews of the controls, each was asked about general well being, the presence of illness, consultation with physicians, and medical tests they underwent both routinely (e.g., work or driver license eligibility tests) or to help diagnose or monitor medical issues. Diagnosis of physical illness was reported using a structured form.

### Data analysis

Lifetime prevalence for DSM-IV major depressive disorder, bipolar disorder, panic disorder, and “at least a diagnosis of anxiety by DSM-IV” was calculated for the case and control groups, as was lifetime positivity (a score >12) on the MDQ as an indicator of bipolar spectrum disorder. The odds ratio association (univariate analysis) for DSM-IV diagnosis (dependent variable) was calculated using the control group as "pivot". Statistical significance was calculated using the *χ*^2^ test in 2 × 2 tables. Odds ratio 95% confidence intervals (OR 95% CI) were calculated using the method of Miettinen [31]. The comparisons between the scores at SF-12 in the study groups were calculated using the ANOVA one-way statistic, in alternative, particularly when the size of the samples did not allow the use of parametric test, the U Mann Whitney test was used.

### Ethics

Each study subject provided informed consent for the use of anonymous data for an aggregate study. The community study (involving the controls) was approved by the ethics committee of the Italian National Health Institute (Rome), and the present study was approved by the ethics committee at the Università Europea del Mediterraneo ONLUS. Data were not nominal at the source, and each subject was identified with a code number.

## Results

The demographic characteristics of the study subjects are shown in Table 1. Age and sex were homogeneous in cases and controls due to the matching method. The mean time on illness for WD patients was about 12 years. Patients with WD had lower SF-12 scores than controls (Table 2), a higher lifetime prevalence of manic/hypomanic episodes (with positivity as assessed on the MDQ), and a higher prevalence of lifetime DSM-IV major depressive disorders and bipolar disorders as detected by the ANTAS interview. The differences between cases and controls in terms of the frequency of panic disorders and total anxiety disorders were notable but not statistically significant: 8.7% vs. 5.4% for panic disorders, and 17.3% vs. 6.5% for all anxiety disorders in cases and controls, respectively.

**Table 1:** Demographic Data and SPECT positivity in the sample

		**Cases (23)**	**Controls (92)**
Age (mean ± sd)		42.02 ± 12.52	42.35 ± 10.29
Age at diagnosis (media ± sd)		30.19 ± 18.28	
sex			
m	9	39.1%	36 (39.1)
f	14	60.9%	56 (60.9%)

**Table 2:** Lifetime Prevalence of Psychiatric Disorders and Quality of Life in Cases and Controls

	**Cases**	**Controls**	**Stat (DF)**	**p**	**OR**	**Cl 95%**
	**(N = 23)**	**(N = 92)**				
SF12	33.76 ± 9.0	38.14 ± 6.4	F(1,113,114) = 7.34	0.008		
MDQ	9 (39.1%)	9 (9.8%)	*X*2 = 4.88 (1)	0.002	5.9	1.3–27.1
Lifetime MDD	11 (47.8%)	11 (11.9%)	*χ*2 = 13.07 (1)	0.001	6.7	2.4–17.3
Bipolar Disorder	7 (30,4%)	3 (3.3%)	*χ*2 = 13.86 (1)	0.001	12.9	3.6–46.3
Panic Disorder	2 (8.7%)	5 (5,4%)	*χ*2 = 0.10 (1)	0.922	1.7	0.1–48.1
Total Anxiety Disorder	4 (17.3%)	6 (6.5%)	*χ*2 = 1.54 (1)	0.215	4.5	0.41–48.4

Table 3 compares Subjective Quality of Life (measured as SF-12 score) in WD patients with Bipolar Disorders (N = 7) – column left; and MDD (N = 11) column right; WD with Bipolar Disorder patients had SF-12 mean score worse than WD patients without Bipolar Disorders (N = 16, 11 of which had MDD) [F (1,21,22 df) = 4.75, P = 0.041 ANOVA 1 way]; had a worse mean SF-12 of WD patients without MDD or Bipolar diagnosis (23WD – 11 (MDD) – 7 (BD) = 5 cases) but the difference not reach the statistically significance [U = 8, Z = -1,54, P = 0.159, using Mann-Withney test due to the not applicability of parametric test]; had SF-12 mean score worse than WD patients with MDD (N = 11), [F(1,16,17) =3.31, P = 0.087, Anova 1 way]. WD with MDD patients had SF-12 mean score higher than WD patients without MDD (N = 12, 7 of which had BD), but without any statistically significance [F(1,21,22) = 0.43, P = 0.519, ANOVA 1 way]; had a worse mean SF-12 of WD patients without MDD or Bipolar diagnosis (23WD – 11 (MDD) – 7 (BD) = 5 cases) but the difference not reach the statistically significance [U = 24,Z = -0,40, P = 0.74, using Mann-Withney test due to the not applicability of parametric test];

**Table 3:** Quality of Life (SF-12 mean ± standard deviation score) and Mood Disorders in Wilson’s Diseases Patients

	**Bipolar Disorders (N = 7)**	**MDD (N = 11)**
	**SF12 = 27.5 ± 7.1**	**SF-12 = 34.4 ± 8.2**
Non Bipolar (N = 16)	F(1,21,22 df) = 4.75	
SF-12 = 36.59.8	P = 0.041	-------------------
Non MDD (N = 12)	-----------------	F(1,21,22) = 0.43
SF-12 = 32.1 ± 9.0		P = 0.519
Non MDD or BD (N = 5)	U = 8, Z = -1,54,	U = 24,Z = -0,40
SF-12 = 37.6 ± 7.6	P = 0.159	P = 0.74
Bipolar Disorders (N = 7)	-------------------	F(1,16,17) =3.31
SF12 = 27.5 ± 7.17.1		P = 0.087
MDD (N = 11)	F(1,16,17) =3.31	--------------------------
SF-12 = 34.4 ± 8.2	P = 0.087	

## Discussion

This study is the first to use a standardized epidemiological case–control design and standardized diagnostic tools (DSM-IV SCID-ANTAS) for studying the association between mood disorders and WD. The results indicated that the lifetime prevalence of DSM-IV bipolar and major depressive disorders is higher in people with WD than in sex- and age-matched controls. The OR was 12.9 for bipolar disorders and 6.7 for major depressive disorders. Notably, there was a difference in prevalence even though (due the random hazard method) the randomized control sample had a particularly high prevalence of bipolar and major depressive disorders than the population from which it was drawn [22]. For example, MDQ positivity was 3.0% in the database population, but 9.8% in the random control sample. These differences were in part due to the fact that MDQ frequencies are highly influenced by sex, age and, in particular residency, and a high frequency in the control group is likely because of these variables. While the controls had a higher prevalence of MDQ (and BP detected by SCID-ANTAS thus, with another method) than in the population from which it was extracted, cases of WD had higher prevalence of MDQ + and BP-DSMIV at statistically significant level than the controls.

According to the algorithm of DSM-IV MDD diagnosis excludes BP and vice versa, and 11 (47.8%) cases had MDD and 7 (30.4%) had BD. The percentage of cases with depressive disorder is in the same range as previous studies cited in the introduction (30–60% for depression as noted in the review of Akil and Brewer [13]). In general, the previous case series showed about 60–80% of comorbidity between WD and psychiatric disorders [17], similar to our data considering that out of 4 cases of Anxiety Disorders, 1 had co-morbidity with MDD and 2 had comorbidity with PD, and only 1 was independent of the mood disorder. Thus, the total psychiatric cases were 19 (82.6%).

The association with bipolar spectrum disorders was confirmed using this more inclusive method, i.e. the MDQ questionnaire, which is capable of including a wider spectrum of bipolar disorders in the “positives” compared to the DSM-IV criteria. The reliability of the diagnosis of Bipolar Disorder using structured tools and the clinical judgment in a clinic is in fact controversial, with some researchers viewing them as reliable [32,33] and others considering them as not reliable [20,34].

Our study shows an association between Bipolar Disorder and WD using the strict algorithm of DSM-IV or using the more comprehensive “spectrum” subject detected by MDQ.

Our study showed an association between bipolar disorders and lower quality of life as measured using SF-12. However, the design of the study did not allow us to clarify whether the bipolarity caused the lower quality of life or whether this was a confounding factor due to another factor (as cerebral damage) causing both bipolarity and lower quality of life.

The strong association between bipolar disorders and WD should be interpreted, keeping in mind the modification of the diagnostic criteria and the evolution of the concept of “Bipolar Spectrum Disorders” in the last few decades [35]. It seems likely that literature reports in the past of “schizophrenia-like psychosis” in WD were due to use of a different definition of schizophrenia compared to current psychiatric diagnostic criteria. In contrast, the association of WD and bipolar disorder may explain the frequent descriptions of “loss of emotional control, hyperactivity, or loss of sexual inhibition, irritability” reported in WD patients [4,16]. A high risk of bipolar disorders in WD may also be related to the higher incidence of suicidal behavior, which is present in 4% to 16% of patients with WD across studies [13,36].

Machado and colleagues [16] suggested that there was a higher association between bipolar disorder and WD than has been reported in the literature. In their case series, which describes the neurological manifestations of 119 patients with WD, the authors observed the psychiatric disorders catatonia, agitation, aggression, delusional thoughts, and mania. The same group, noting that there are few reports in the literature of WD patients with typical bipolar affective disorder [18], described a patient with WD whose initial manifestation was a manic episode followed by depression. Tremors in the upper limbs appeared one year after the onset of symptoms. The diagnosis of WD was established three years after the first symptoms appeared, based on the neuropsychiatric picture, detection of Kayser-Fleischer rings, and the results of diagnostic tests that indicated chronic liver disease and excess copper. Another case of mania as the first manifestation of WD was described earlier by Kumar Chand et al. [19] in an 18-year-old boy who presented to a psychiatric clinic with manic syndrome and a high propensity for extrapyramidal symptoms due to a neuroleptic. The author concluded that psychiatrists need to recognize that WD can rarely present as an isolated psychiatric symptom, including mania. Thus, early and severe extrapyramidal symptoms secondary to neuroleptic exposure in an adolescent warrant a detailed evaluation to rule out underlying neuropsychiatric conditions. Our results, which underline the strong association between bipolar disorder and WD, support this idea.

It is interesting to consider these results in light of the pathogenic hypothesis.

Early studies showed that women affected by chronic depression sometimes have copper, zinc, and cesium deficiencies [37,38]. Later studies suggest that the presence of depression and other neuropsychiatric symptoms is due to the deposit of copper in the central nervous system [39]. Eggers et al. [40] used SPECT to demonstrate a reduction in thalamic-hypothalamic presynaptic dopamine and serotonin transporters due to the accumulation of copper. There was a negative correlation between the density of presynaptic dopamine transporters and the severity of depression as assessed using the Hamilton Rating Scale for Depression.

It was recently hypothesized that trace elements play an important role in the pathogenesis of bipolar disorders by causing neurodegeneration [41]. Moreover, essential elements like vanadium have been implicated as a causative factor for bipolar mood disorder, while elevated vanadium and molybdenum levels have been reported in serum samples from bipolar mood disorder patients [41]. This latter study showed, using DSM-IV standard diagnostic criteria and classification into types I, II, and V according to the concept of Young and Klerman, that Na, K, P, Cu, Al, and Mn were elevated significantly in Bipolar I (Mania) (P < 0.001). In Bipolar II hypomania, Na, S, Al, and Mn were increased significantly (P < 0.02), while in Bipolar II depression, Na, K, Cu, and Al were increased significantly (P < 0.001). Finally, in Bipolar V, Na, Mg, P, Cu, and Al were increased significantly (P < 0.002) compared to a control group [41]. A recent study by Gonzales-Estecha and colleagues [21] found higher serum copper and zinc, blood lead and cadmium, and urine lead, cadmium, and thallium concentrations in patients diagnosed with bipolar disorders compared to a control group.

Increased neuronal oxidative stress (OxS) induces deleterious effects on signal transduction, structural plasticity and cellular resilience, mostly by inducing lipid peroxidation in membranes, proteins and genes [42]. It has been hypothesized that these pathological processes occur in critical brain circuits that regulate affective functioning, emotions, motoric behavior and pleasure involved in bipolar disorder (BD) [43,44].

The brain is particularly vulnerable to oxidative damage since it contains large amounts of polysaturated fatty acids and possesses low antioxidant capacity [45]. Several studies have demonstrated altered OxS parameters in the pathophysiology and therapeutics of BD, including changes in the levels of enzymes superoxide dismutase (SOD), catalase (CAT) and thiobarbituric acid reactive substances (TBARS) [46]. The well-known stabilizing agent Lithium was found to limit the enzyme activity, potentially lowering hydrogen peroxide and hydroxyl radical formation. Similarly, lithium was also shown to reverse increased OxS parameters in BD [43,47]. For instance, a decline in lipid peroxidation and an increase in CAT levels were observed in valproate and lithium-treated rats [42,48]. Accumulation of copper was shown to increase oxidative stress in bivalve species [49]. In skeletal muscle of Broilers Under Heat Stress, copper decreases because of dietary Selenium, Vitamin E, and their combination with an increase in antioxidant defense [50]. In humans accumulation of copper was associated with oxidative stress in allergic asthma patients, and introduction of nutritional supplement therapy accompanies improved oxidative stress, immune response, pulmonary function, and decrease in copper plasma levels [51]. On the other hands copper levels were elevated in several brain areas in a degenerative disease such as Niemann-Pick C [52]. Interestingly, Nieman-Pick C disease was specifically indicated to be associated with Bipolar Disorders [53]. If the results of our study are further confirmed, it will lend significant support to the hypothesis that minerals such as copper play an etiological role in psychiatric disorders, and WD may serve as a pathogenic model for the bipolar disorder.

## Limitations

This study was limited by its small sample size. Further research is warranted to confirm the results and the role that neurological lesions play in the pathogenesis of bipolar disorders. In addition, the link between neurological lesions, bipolar disorders, and low level of quality of life in WD awaits further clarification.

In agreement with the literature [11], this study indicated a high risk for panic disorder (OR = 1.7) and for anxiety disorders as a whole (OR = 4.5) in WD, although there was no statistically significant difference between cases and controls. The finding that WD patients did not have an increased risk of anxiety disorders must be interpreted with caution since the power of the study was insufficient in terms of revealing low-level risk.

In this study, the diagnosis of WD in the cases was made using standardized tools and clinical and laboratories assays. In contrast, the data bank used for extraction of control data reported a WD diagnosis only on the basis of the anamnesis of the subject in the ‘general health’ section of the questionnaire. Thus, there may be a slight risk of false negatives in the group of controls, although this risk was very low due to the rarity of WD in the community (1:30,000; thus the probability of a control subject not reporting WD would be 30,000/92). Even if there was a false negative among the control subjects, this would reinforce the null hypothesis of no increased risk for mood disorders in WD. This limitation therefore does not invalidate the results of the study.

In general, reliability and validity of diagnosis of Bipolar Disorders using standardized tools (as ANTAS-SCID) and self-administered questionnaire (as MDQ) [18], may be problematic, however, this is a general limitation of the epidemiological research in psychiatry more than a specific limit of this study. We have tried to address this limitation, at least partially, by using a clinician for the interview and a semi-structured tool as opposed to a medically lay person with rigid and structured interview and by using both methodologies (interviews and self-reporting tools).

This study may have inducted a Berskon’s Bias due to the case control design, on the other hand, the study was carried out in a general hospital setting without a psychiatric ward, therefore this risk is relatively low.

## Conclusions

This study was the first to show an association between BD and WD using standardized diagnostic tools and a case control design. Reports in the literature about increased schizophrenia-like psychosis in WD and a lack of association with bipolar disorders may thus have been based on a more inclusive diagnosis of schizophrenia in the past. Our findings may explain the frequent reports of loss of emotional control, hyperactivity, loss of sexual inhibition, and irritability in WD patients.

## Competing interests

The authors declare that they have no competing interests.

## Authors’ contributions

MGC participated in the design and coordination of the study, in the acquisition and analysis of the data and drafted the manuscript. MP participated in the analysis of the data and drafted the manuscript. LD participated in the design and coordination of the study and critical revision of the manuscript. KMB participated in the analysis of the data and drafted the manuscript. MFM, OS, ED, AS, GM, FS participated in the design of the study, in the acquisition and analysis of the data and drafted the manuscript. All authors read and approved the final manuscript.

## Pre-publication history

The pre-publication history for this paper can be accessed here:

http://www.biomedcentral.com/1471-244X/12/52/prepub
